# Doing respectful closeness: the role of minimal listening responses in supporting disclosure of sexual offenses

**DOI:** 10.3389/fpsyg.2026.1720312

**Published:** 2026-09-28

**Authors:** Mari Todd-Kvam, Anja Emilie Kruse, Nicolay Gausel

**Affiliations:** 1 Department of Social Work, Uppsala University, Uppsala, Sweden; 2 Norwegian Centre for Violence and Traumatic Stress Studies (NKVTS), Oslo, Norway; 3 Institute of Psychology, University of Stavanger, Stavanger, Norway

**Keywords:** interactional analysis, intersubjectivity, longitudinal conversation analysis, relational work, sexual violence perpetration, unconditional accept

## Abstract

**Objective:**

Across institutional settings, there are conversations in which the client’s disclosure of moral transgressions is a goal in itself or a necessary step toward change. Such disclosure both depends on and may threaten the client–professional relationship. Therefore, these conversations constitute a fruitful setting for examining how people build purposive relationships within institutional constraints. We aim to examine how participants interactionally co-achieve a relationship that is purposive toward disclosure of moral transgressions.

**Methods:**

Drawing on conversation analysis (CA) of 19 research interviews with young people who have committed sexual violation(s), we apply longitudinal CA on one single successful case, substantiated by three contrastive examples.

**Results:**

Our analysis demonstrates how a purposive relationship quality of respectful closeness is co-achieved through the interviewer’s responsive and skillful use of minimal listening tokens to support the interviewee’s progression toward disclosure. We specify one overarching dynamic (mutual trust) and three practices (staying with difficulty, relationally scaffolded storytelling, and managing absence with presence) characterizing this relational work.

**Conclusion:**

Relationship quality is achieved on a turn-by-turn basis and may over time result in the relational robustness necessary to disclose sexual offenses. Conversation analysis detailing this relational work can develop both theory and clinical practice, and unpacks expertise as practical, observable knowledge.

## 1 Introduction

The interaction between client and professional is pivotal in enabling clients to address their distress and work toward change (Muntigl, 2020). While the association between client–professional relationships and positive outcomes is well established (Wampold, 2015), a “next generation of questions” (Muntigl and Horvath, 2014, p. 327) asks how such purposive relationships are built and maintained. Answering these “next questions” benefits from an *interactional* approach: examining how participants in clinical conversations build on and resist each other’s turns of talk to accomplish core relational processes such as finding common ground or managing rupture and repair (Muntigl and Horvath, 2014; Muntigl et al., 2013; Scarvaglieri, 2020; Voutilainen et al., 2010b; Weiste and Peräkylä, 2014). The interactional perspective enables us to observe how people position themselves relative to each other through what they do with their talk. Through detailed examination of the relationship as an interactional process, this strand of research thus demonstrates how “we do not simply “become” close or distant: it happens turn by turn, moment by moment” (Stivers, 2022, p. 21).

This turn-by-turn co-creation of the relationship is highlighted in settings where achieving the conversation’s purpose depends on participants’ capacity to address topics that may threaten their relationship. Sexual violence perpetration is one such topic, being widely condemned and engendering public discourses that pathologize and demonize those who perpetrate it (Kruse and Houge, 2025; Kruse and Skilbrei, 2024). Disclosing severe moral transgressions such as sexual offenses therefore involves very real risks to social belonging (Gausel and Leach, 2011)*—*both in society through shaming (Ahmed et al., 2001), and in interpersonal relationships (Ellemers et al., 2008; Gausel, 2023). Therefore, while the topic may be both experienced (Blagden et al., 2011) and treated (Auburn, 2005) as threatening the relationship, the quality of this very relationship is paradoxically also a necessary condition for approaching morally sensitive topics (Grönte et al., 2024; Prior, 2018; Rogers, 1961; Silverman and Peräkylä, 1990).

This focus on the relationship is mirrored in the broader literature on institutional responses to moral transgressions, which converge on a relational ideal characterized by unconditional acceptance (Rogers, 1961). Within both mental health and criminal justice services, the simple “how can I be of help”–style of conversation is assumed necessary and sufficient to create a trusting relationship where morally sensitive topics can be approached, and institutional agendas of rehabilitation and repair can be pursued (Ahmed et al., 2001; Braithwaite, 1989; Brewer and Giommi, 2025). Interactional research on addressing morally sensitive topics show how participants often design their talk to signal awareness of the potentially unsociable nature of these topics (Silverman and Peräkylä, 1990) and spend time doing preparatory work *before* they address them (Iversen et al., 2017). In institutional talk about sexual offences, professionals accomplish relational skills such as warmth and respect (Mullins and Kirkwood, 2022) and participants manage specific challenges such as neutralizations (Auburn, 2005; Todd-Kvam et al., 2025) and expressions of self-reproach (Mullins and Kirkwood, 2019) in ways that enable participants to approach and stay with the topic.

This research demonstrates how institutional conversations about moral transgressions, including sexual offenses, highlight relational ideals relevant beyond moral transgressions, to relational work in clinical settings. For instance, psychotherapy research has examined the Rogerian ideal of patient-centered therapy (Muntigl et al., 2013) and the management of client self-criticism (Muntigl et al., 2020) as interactional achievements.

However, while psychological and criminological research describe relationship quality in terms of the experience it should evoke in the person disclosing (being unconditionally accepted), it fails to clarify how participants co-create this relationship quality through their turn-by-turn interaction. And while interactional research has unpicked specific challenges in institutional conversations about sexual violence perpetration, it has hitherto not examined the co-achievement of relationship quality *itself*. We therefore need more research into *relationship quality as a continuous interactional achievement*. This knowledge need is addressed in psychotherapy research (Muntigl and Horvath, 2014) and in interactional research on relationship-building, where “more work is needed to identify and explain how these elements are jointly realized, discursively, and how a certain relationship quality is achieved and maintained in the process” (Muntigl and Scarvaglieri, 2023, p. 6). Therefore, this article examines how participants over time build and maintain a relational quality purposive to addressing a topic that may threaten this very relationship.

Previous interactional research has described qualities of the relationship as “social bonding and cooperation” (Etelämäki et al., 2021, p. 727), “harmony, empathy and cooperation” (Muntigl and Horvath, 2014, p. 329), and “solidarity” (Clayman, 2002; Heritage, 1984; Lindström and Sorjonen, 2013; Pino, 2022). Following this approach, we do not aim to establish what a good relationship *is* in principle, but to unpack *how* participants successfully achieve a relationship that is purposive (or not) to stakes and agendas *in their local interactional setting*. We provide a detailed, in-depth analysis of relational work done toward the purpose of facilitating disclosure of sexual violations, in research interviews with young people. In addition to being frequently used within qualitative psychology research and clinical work, interviews are particularly suitable for unpacking key aspects of relational work within institutional settings.

## 2 The interview as a setting for examining relational work

In interviews, participants’ relational work is done *within* institutional constraints on the relationship-building strategies they may use, and *while* pursuing the interview’s agenda. Interactional research demonstrates how interviewers balance their role and the structure of the interview on one hand, against everyday conversational norms and concerns with relationship building on the other (Arminen and Simonen, 2021; Houtkoop-Steenstra, 2000; Iversen, 2018; Prior, 2018). This is particularly acute in interviews about morally sensitive topics. Many resources for attending to the relationship, such as sharing or normalizing the interviewees’ problems, providing assessments and moving blame from the interviewee to their context (Houtkoop-Steenstra, 2000) are here potentially problematic. Interviewers may be caught between ideals of neutrality, the agenda of facilitating further talk, and an imperative against endorsing problematic content (Todd-Kvam et al., 2025; Zhang and Okazawa, 2023). These challenges are relevant not just when interviewers produce full turns (such as questions), but in how they convey *listening*. Even minimal listening responses may do secondary actions (Levinson, 2013) in addition to conveying listening, such as displaying (lack of) uptake (Sikveland, 2012), steering (Sikveland et al., 2021; Todd-Kvam et al., 2025), or undermining (Lee and Bhuyan, 2013) interviewees’ ongoing talk. These secondary actions are often achieved with subtle nuances in prosodic design (Ogden, 2006; Sbertoli-Nielsen, 2023; Sikveland, 2012; Sikveland et al., 2021), which may enable interviewers to convey relational support within the institutional constraints of their role by being off-the-record (Iversen et al., 2024; Stivers et al., 2023). As such, interviews constitute unique and useful settings for examining relational work within institutional constraints. Specifically, using verbal and non-verbal nuances in minimal listening responses to build a purposive relationship with the interviewee constitutes *expertise* as a form of practical knowledge (Arminen and Simonen, 2021; Risjord, 2015), and unpacking this expertise, turn-by-turn, can enrich knowledge about, and training for, interviews in general (Richards, 2011; Roulston, 2006).

## 3 Materials and methods

### 3.1 Materials

The material comes from a multi-disciplinary research project on sexual violence in young people’s intimate relationships run between 2020 and 2024.^1^ Our analysis draws on data collected as part of this project: 19 audio-recorded interviews, conducted across Norway with young people who have committed sexual violations. Interviewees provided informed consent, and to avoid creating an incentive to consent economic compensation was not given. Interviews lasted 60–90 min, resulting in a total of 1,500–2,000 pages of verbatim transcribed material. Interviewees (17 men, 2 women, ages 18–25) were recruited through the prison officers working their cases or via social media, as convicted of a sexual violation or self-identifying as having committed one. Interviewers had substantial experience doing research interviews on sensitive topics, some specifically with the topic of violence perpetration. This analysis’ main extract (Extract 4) was conducted by an expert interviewer, with several years of experience in doing research interviews with victims and those perpetrating sexual and domestic violence. Before and during interviews, interviewers carefully considered ethical issues such as process consent, interviewer safety, and interviewees’ wellbeing (Pascoe Leahy, 2022). All participants were offered a follow-up session with a psychologist specialized in sexual violence; only a few requested it. The Norwegian Agency for Shared Services in Education and Research (SIKT) approved the project’s strict data storage system, and relevant regional offices of the Norwegian Correctional Service approved its ethical implementation. The current analysis was also approved by a Swedish Regional Ethics Committee.

### 3.2 The specific interview setting

The research project’s original agenda with the interviews was to get interviewees’ narratives about having committed sexual violation(s), to then analyze interviewee answers from social work, criminological and sociological perspectives. Interviews were therefore conducted with the aim of eliciting rich interviewee’s stories, so interviewers took a neutralistic stance aimed at facilitating disclosure without steering its content (Rapley, 2001). Interviewees, who were recruited based on belonging to a morally condemned category (persons who have committed sexual violations), likely had an interactional stake in accounting for their actions (Potter and Hepburn, 2005; Rapley, 2001). Despite the interviews being limited to one or two encounters, and involving a professional, not personal relationship, these stakes and agendas challenge the participants as they pursue the goal of getting rich stories about interviewees’ experiences. This pursuit involves managing a shared expectation of the topic as relationally challenging (Auburn, 2005; Blagden et al., 2011) and interactional challenges the topic invokes (Auburn, 2005; Mullins and Kirkwood, 2019; Todd-Kvam et al., 2025). The combination of high social-relational stakes and interviewers’ stake in gaining interviewees’ stories made these interviews particularly suitable for the focus of this current analysis: how participants interact, turn-by-turn within an institutional setting, to build and maintain a quality of their relationship that enables disclosure of morally sensitive topics.

### 3.3 Analytic approach and terminology

We take a conversation analytic approach (Sidnell and Stivers, 2013), examining what the participants do (actions and practices), what resources (such as word choice or voice quality) they use to achieve these actions, and how these actions respond to preceding actions and constrain subsequent ones. We draw on this to analyze how people position themselves relative to one another through their turns of talk, and thus how they “do” their ongoing relationship (Stevanovic and Peräkylä, 2014; Stivers, 2022).

People may display and achieve the momentary quality of their relationship over a single pair of turns (e.g., by how one greets one another; Pillet-Shore, 2012) but also negotiate who and what they are to one another over *multiple* turns of talk (Etelämäki, 2021). To analyze how interviewer and interviewee come to accomplish the interviewee’s disclosure together, we therefore use longitudinal conversation analysis (Deppermann and Pekarek Doehler, 2021). This approach enables us to examine participants’ interactional history—how they “produce joint action by which shared knowledge and reciprocal expectations of action and understanding develop” (Deppermann and Pekarek Doehler, 2021, p. 131). In the following, we outline some conversation analytic concepts that “help us gain analytic leverage to understand what speakers are doing relationally” (Stivers, 2022, p. 3) with their turns of talk.

*Alignment* involves “getting in line” with the action or activity proposed (Steensig, 2020; Stivers, 2008). Alignment is a structural aspect of the conversation, that can be used to achieve relational outcomes such as collaboration and common ground. *Affiliation* describes actions that support, endorse, or respond empathically to a display of emotion or an emotional stance, or respond with the socially preferred action (Steensig, 2020; Stivers, 2008). Affiliative actions display that you are “with” someone (Lindström and Sorjonen, 2013, p. 355), “mitigating threats to solidarity” (Lindström and Sorjonen, 2013, p. 368).

We analyze both *how* participants affiliate and align and *what they orient to* in the previous turn. Here, and Stevanovic and Peräkylä (2014) nuanced treatment of the momentary relationship as claimed and demonstrated within three domains of the interaction—the epistemic, the deontic, and the emotional—is useful (see also Enfield, 2011).

In brief, we analyze closeness (and distance) as orienting to shared understanding (or not) within the epistemic domain, emotional intimacy (or not) within the emotional domain, and shared decisions (what to talk about, who gets to ask questions), activities (storytelling) or projects (arriving at a meaningful story), or not, within the deontic domain. We analyze autonomy as how the speaker’s independency of action (Stivers, 2022) is claimed and respected interactionally: what they can expect to be treated as more knowledgeable about (epistemic), to what extent they are allowed to decide on interactional matters (deontic) and to what extent they are allowed to express their emotions (Stevanovic and Peräkylä, 2014).

### 3.4 Analysis

We analyze each turn of talk in terms of what it responds to and what it makes relevant for the subsequent talk, continuously checking our understanding of a turn against how it is treated by the participants in the subsequent turn (Sidnell and Stivers, 2013). In line with our focus on the relationship as co-created *in* and purposive *to* the specific setting, we here examine relationship quality as relevant to the purpose of enabling interviewees’ disclosure. In the preliminary analysis (all 19 interviews), it became clear that relational work is done through a range of different verbal and non-verbal resources, and often with minimal turns, especially when participants addressed the interviewees’ sexual violations (as opposed to questions about socio-demographic factors). Such sequences were therefore transcribed using Jefferson (2004) annotation system and, in a second round, the more phonetically detailed GAT-2 annotation system (Couper-Kuhlen and Barth-Weingarten, 2011). To ensure readability for readers not versed in the specific annotation system of conversation analysis, we present the findings with selected Jeffersonian annotation symbols (Table 1). In the main extract, GAT-2 annotation is presented in italics for the interviewer’s minimal responses, and original language is given in footnotes for turns where important meaning may get lost in translation.^2^

**Table 1:** Selected annotation symbols after Jefferson (2004).

Annotation	Meaning
CAPITAL LETTERS	Louder volume
Emphasis	Stress on marked syllable
Some [words] in brackets [overlap]	Overlapping talk
°word°	Lower volume, sotto voce
(.) / (1.0)	Micropause/1s pause
.	Turn-final intonation: downwards
,	Turn-final intonation: slight rise
?	Turn-final intonation: sharp rise
_	Turn-final intonation: flat
Wor-	Cut-off word
Wo::rd	Elongated word
turn= =turn	Latch-on (the turn comes immediately after the previous)
↑/↓	Marked rise/fall in pitch

As the analysis progressed, we became interested in how participants used different resources *over multiple turns of talk* that together built and maintained the quality of their relationship. We therefore chose to present one longer section of talk “from the beginning without a gap” (Deppermann and Pekarek Doehler, 2021, p. 132) in order to enable “not only the identification of interactional patterns but also the discovery of how one instance of interaction may lead to a next” (Deppermann and Pekarek Doehler, 2021, p. 123, see Kupetz, 2014; Voutilainen et al., 2010a for examples).^3^ We support our analysis with contrastive examples from sequences where disclosure came to a halt. Our unit of analysis thus became the section as a whole as well as the practices within it. We propose these practices *do* a specific relationship quality that enables disclosure, evidenced in the achieved disclosure in this section (ex.4) and in how the same practices observably are not achieved in contrastive examples (ex-1-3).

## 4 Results

Our main finding is how interviewer and interviewee (Extract 4) co-achieve a subtle and multi-layered relational quality of *respectful closeness*. They achieve this respectful closeness over multiple turns, in a dynamic where the interviewee (Kristoffer) contributes by treating his difficulties in disclosing as something they can potentially manage together, and the interviewer responsively orienting to this by both respecting Kristoffer’s autonomy in deciding how he approaches the topic and conveying continued closeness within the institutional constraints of her role.

The participants achieve this relationship quality using a diversity of subtle resources and off-the-record secondary actions (Levinson, 2013). To cue the reader to this subtlety, we first present three contrastive cases. In these cases of momentarily disrupted or derailed disclosure, the relational closeness is lacking or boundaries are overstepped. Thereafter, we show our longer disclosure sequence to demonstrate practices for co-achieving respectful closeness in the face of relational challenges.

### 4.1 Three cases of disruption

Both interviews where rich disclosure was achieved and those where it was not contained sequences of talk where the participants’ ongoing work toward disclosure was momentarily ruptured. In our clearest example, we meet Tord, who is interviewed while serving a prison sentence. We come in toward the end of the interviewer’s lengthy pursuit of Tord’s reflections on his violations, where she has just asked him about his thoughts:

**Extract 1: Not taking interpersonal risks***(IE = interviewee, IR = interviewer)*07	IE:	na:::h thinking it’s a waste of time, =to
08		sit here?
09			(1.0)
10	IR:	↓yes.
11	IE:	so (.) wouldn’t have minded not doing it
12	IR:	yes_
13			(1.0)
14	IR:	but would you say you understand why15		or?=why you were convicted?
16 			(.)
17	IR:	or?
18 			(.)
19	IE:	yeah I have been told, you know, that it’s not okay20		*da*,=but like i::t (.) is not something I walk around
21		thinking about like_

In Tord’s answers to the interviewer, we can note three things obstructing a close relationship, with a resulting lack of disclosure. First, he displays a lack of investment in the matter (lines 7–8 and 11). Second, he demonstrates awareness of the dominant social and legal view, but disavows it (lines 19–21), thus rejecting his accountability and misaligning with the interview’s pursuit of *his* reflections (lines 14–17). Finally, his indifferent emotional stance robs the interviewer of resources to achieve emotional closeness, unless she affiliates with his implicit critique of society’s view on sexual violence. In the continued talk (not seen in the extract), he goes on to display his recognition of the interviewer’s pursuit of his reflections and stance display, and rejects that, too (“I don’t have any compassion for her [the victim], if that’s what you’re wondering about”).

In our second example, it is the interviewer’s responses that disrupt a close relationship, thus derailing disclosure. We come into the interview immediately after the interviewer has asked Adam, who is serving a prison sentence, what he thinks his victim “feels happened”:

**Extract 2: Not doing it together**77	IE: 	no for sure that e::h (1.5) it came as a bit of a surprise
78		on her?
79	IR:	mm_
80		(.)
81	IE:	that she wasn’t as ready as sh- (1.0) as I82		thought?
83	IR:	mm_
84		maybe?
85		mm_
86		(2.0)
87	IE:	(unclear) a little kind of misunderstood there?
88	IR: 	mm_
89		(2.0)

Adam’s candidate answers (lines 77–78, 81–82, 87) put the interviewer in a difficult position: by being designed as tentative, with a upward turn-final intonation, they make her co-reflection relevant. However, co-reflecting would challenge the interviewer’s neutralistic stance and possibly be morally problematic: the candidate answers can come across as minimizations (lines 77–78 and 87: misunderstanding, surprise) and victim-blaming (line 81–82). The interviewer’s responses (lines 79, 83, 85, 88) work to disrupt closeness not just by misaligning (i.e., not co-reflecting) but also by *not* taking any other measures to orient to the relationship (such as designing her disaligning responses as dispreferred; Pomerantz and Heritage, 2013) and not using other resources to repair (Muntigl and Horvath, 2014; Muntigl and Scarvaglieri, 2023). These unmarked listening responses resist common ground while not explicitly misaligning, giving Adam a chance to back down (Iversen et al., 2024). In combination, they signal non-uptake of Adam’s interactional project of co-reflection, by virtue of being verbally and non-verbally identical (Sikveland, 2012).

In our final example of disruption, we meet Hans who, earlier in the interview, has described committing a rape. We come in after Hans and the interviewer have engaged in a shared project of reflecting (i.e., intellectually) on Hans’ crime from a socio-cultural perspective, as the interviewer asks if Hans was sexually turned on by the power:

**Extract 3: Disalignment**156	IE: 	so I think [that]
157	IR: 	                [did it] turn turn you on kind
158		of?=that you- that that power kind of?
159	IE: 	e:::::[:h]
160	IR:	        [o]r was it just that it was
161		more comfortable well I’m just wondering jus::t_
162	IE:	eh hhhh well I don’t kno:::w exactly .hhh i::n
163		just in the moment then it ↓was e::h I think
164		mohhre adrenaline than anything.165		    (.)
166	IE:	bu::t I think the adrenaline too comes from that
167		(1.0) experience of power and ↑control though.

Here, neither the interviewer’s nor the interviewee’s responses disrupt closeness. Rather, it is a misalignment between the participants’ interactional projects (Levinson, 2013), resulting in overstepped boundaries. The interviewer’s somewhat sudden motion to change focus, from the intellectual to the intimate, both misaligns with their hitherto shared project of intellectual reflection and is misaligned with by Hans’ next turn (line 159). Although the interviewer orients to the rupture and tries to repair by replacing the term with a less sexually loaded word (“comfortable”) and de-weaponizing her intentions (lines 160–61), the subsequent disclosure is thin, perturbated, and impersonal. Hans claims a lack of access to his own experience (line 162: “do not know”), separates the experience from his usual self (line 163: “just in the moment”) and describes it as chemical rather than personal (“adrenaline”). Moreover, he continues to treat his experience as an object of analysis as opposed to his own experience (lines 166–67: “that experience”).

These three examples show sequences in which the institutional goal of the conversation, namely the disclosure of sexual violence perpetration, was momentarily disrupted or derailed. What characterizes them is an absence of respectful closeness: either a combination of misalignment and lack of (opportunities for) affiliation create too much distance (Extracts 1 and 2), or a misalignment of conversational projects results in overstepped boundaries (Extract 3).

### 4.2 Co-achieving a relationship in which disclosure can happen: a longer sequence

In this section, we meet Kristoffer, who is interviewed while serving a sentence for sexual offenses against minors, in a small prison located near Oslo. Throughout the interview, Kristoffer orients to his fear of being rejected as someone who is just excusing themselves—indirectly at the start of this sequence, and explicitly later in the interview (not shown in the extracts). The shown section of Kristoffer’s interview (Extract 4) represents the beginning of a returning overarching dynamic in it: as Kristoffer approaches his offenses, he oscillates between describing and explaining them, and the interviewer follows and supports him through these oscillations. We have named this overarching dynamic *mutual trust* and will describe it further after demonstrating it throughout the extract.

Our example will take us through a question (line 29: “what was it that you did”) that is successfully answered 69 lines later (lines 99-100: so within there then I slept with (.) ↑((anonymized) (see Extract 4.4 footnote a)) who were under ↓sixteen.). To highlight Kristoffer’s initiatives and the interviewer’s responsiveness, we have divided the extract into four phases based on Kristoffer’s overarching interactional projects: approaching *telling*, *commencing disclosure*, *returning to understandability*, and *arriving at the answer*. Within and across these phases, we specifically analyze three practices through which the interviewer’s skillful use of minimal listening tokens responsively attends to Kristoffer’s different ways of making relevant relational support: *staying with difficulty, managing absence with presence* and *relationally scaffolded storytelling* (see Figure 1). For the reader to follow the overarching dynamic of mutual trust, including the transition between phases (Deppermann and Pekarek Doehler, 2021), we show the extract without gaps. While this displayed section of the interview ends up in a mitigated and vague disclosure, the continuous achievement of respectful closeness represented here results in a more elaborate disclosure later in the interview.

**Figure 1:**
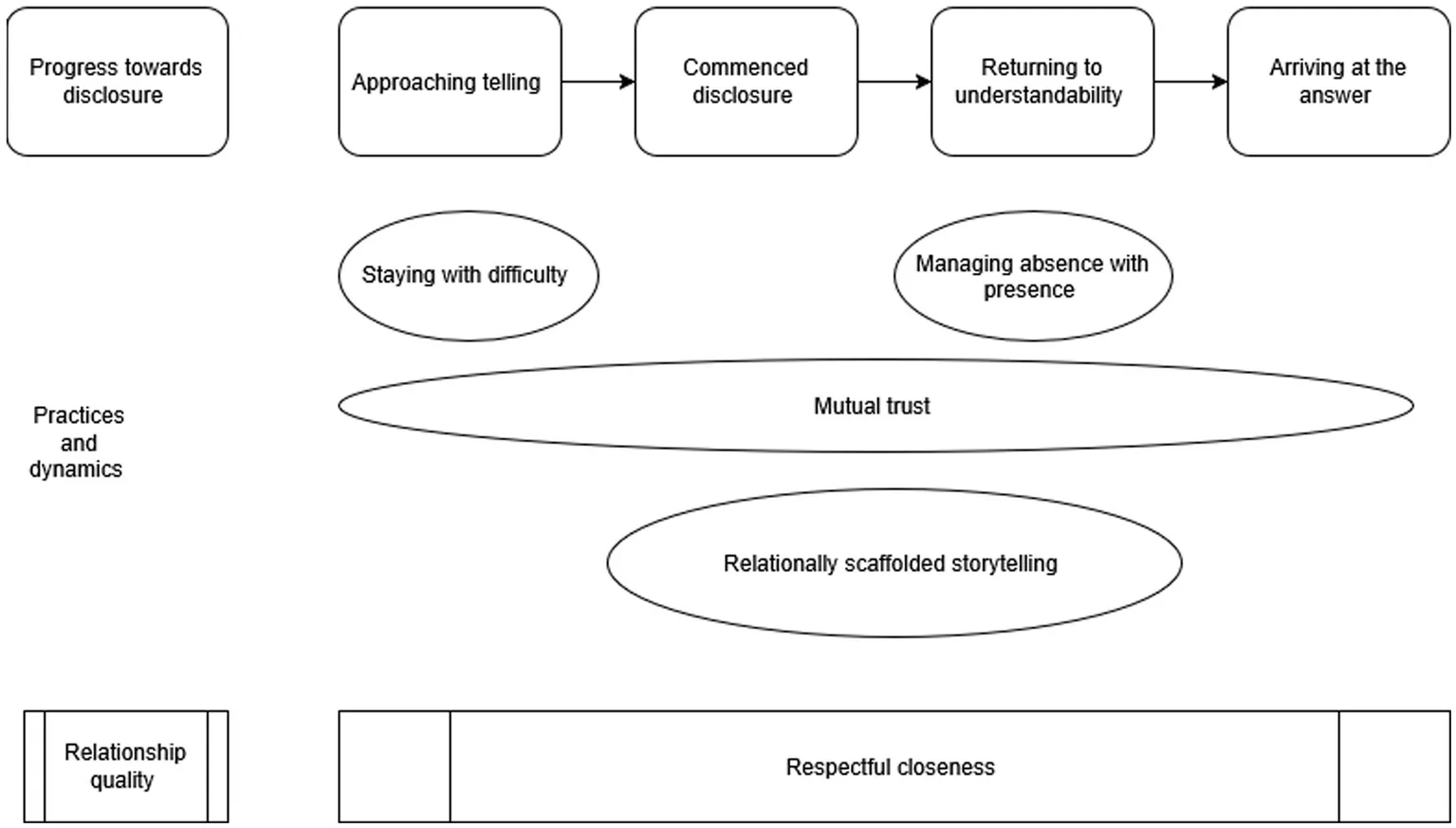
Throughout the extract, the interviewer’s responsive use of minimal listening tokens supported the interviewee’s turn by turn progress toward disclosure. Within this interaction, we specify three practices and one overarching dynamic that all contributed to a co-achieved quality of respectful closeness in the relationship between them.

#### 4.2.1 Approaching telling: displaying difficulty and staying with difficulty

In this phase, Kristoffer and the interviewer set up an interactional space to approach Kristoffer’s disclosure. We highlight one phenomenon and its consequence: the participants’ willingness to stay with what is difficult, and the subsequent space that opens up for them to negotiate the capacity of “the others” to understand (i.e., a threat of rejection). We come in as Kristoffer describes addressing his own victimization experiences to better understand how he came to commit his offenses. “It” refers to this work, which he describes as emotionally hard:

**Extract 4.1a: Approaching telling**01	IE:	but it helps a great deal (.) along the way a::nd
02		(.) especially (.) th-those from the outside that see th-f-
03		kind of “wow he has committed a crime” (.) then
04		it makes it perhaps a bit easie:r for me to (.)
05		explain why this happened_
06	IR:	mm, *(GAT-2: ^ˇ^Mm,)*07			(1.0)

Here, by explicitly addressing his concern that “those from the outside” (line 2) might not understand—without being clear as to whether he includes the interviewer in this category—Kristoffer’s turn addresses the risk of rejection involved in disclosing his offense. Moreover, the turn is rife with hedging (“kind of,” “a bit”) and “perturbations”: pauses, elongated words, and cut-offs. This can be understood both as marking the topic as sensitive and requiring careful handling (Silverman and Peräkylä, 1990) and as an emotional display (cf. Retzinger, 1995). Unlike Tord in our first example, Kristoffer orients to his responsibility to account for his violence, thus rendering the potential judgment from “those from the outside” important to him.

The interviewer’s short response (line 6) works as a *continuer*: it displays listening, but does not take the floor, thus aligning with Kristoffer’s storytelling as ongoing (Schegloff, 2007). Its prosodic design indicates “agreement” (Sbertoli-Nielsen, 2023), while the narrow pitch range indicates lower investment (than a wide pitch range; Ogden, 2006). However, it leaves it ambiguous as to what is acknowledged or (mildly) agreed with. In combination with *not* treating any of the implicit assumptions as in need of repair or clarification (Robinson, 2014), we understand the turn as acknowledging Kristoffer’s concern (over others’ potential rejection) as legitimate and at the same time making irrelevant the interviewer’s position vis-à-vis “the others.” She neither rejects him nor avoids his fear of rejection but *stays* in this interpersonally difficult space. By not attempting to change Kristoffer’s displayed emotion but accepting his assumptions, thus legitimizing such an emotional reaction, the interviewer also stays with the emotional difficulty Kristoffer displays.

By displaying and staying with the interpersonally and emotionally difficult, the participants co-achieve their momentary relationship as one in which there is space to talk about vulnerable and intimate concerns—and where Kristoffer gets to set the parameters of this space. Kristoffer and the interviewer go on to set up some important premises for his disclosure:

**Extract 4.1b: Approaching telling, continued**08	IE:	I don’t think they would understand if I just
09		like “I woke up and it just happened.”
10	IR:	↓no_= (*GAT-2: ↓−nei_*)
11	IE:	=but if one has a bit like (.) background “this
12		is everything you didn’t kn[ow h]appened before it
13	IR:			                  [m:] *(GAT-2: `m:)*14	IE:	happened.”
15	IR: 	mm; *(GAT-2: `mm;)*16	IE:	then they may have a bit more understanding that
17		(.) e:h it’s not that I (.) want to make
18		my case innocent,^a^19	IR:	m:_ *(GAT-2: m:_)*20	IE: 	but (.) e:h (1.0) there was a lot that happened that
21		led to (.5) ↑me committing=
22	IR:	°=m:_=° (*GAT-2: °=`m:_=°*)
23	IE:	=crimes.=
24	IR:	°=m.° (*GAT-2: °=`m.°*)
25	IE: 	that makes it perhaps a bit easier for
26		↓them to manage the things,
27	IR: 	eyeah. (*GAT-2: `eja*.)
28		(1.0)
29	IR:	.hhhh (.) °↑what ↓was it you did then.^b^^a^Original language:  «så har de kanskje litt mer forståelse at (.) e:h det er ikke det at (.) jeg skal gjöre min sak uskyldiggjort,»^b^Original language: «.hhhh (.) °↑hva ↓var det du gjorde da.»

The first premise they set up is that while he can fear others’ lack of understanding (lines 8–10), it is *possible* for others to understand (line 16). The “problem” of others not understanding is treated as an issue of insufficient knowledge (lines 11–14), but with relational undertones (“understanding” in line 16 may mean both comprehension and acceptance in Norwegian). The interviewer’s listening responses responsively varies with Kristoffer’s talk, both lexically (an acknowledging “no” to his statement in line 10, and variations of “m” thereafter doing continuers as their main actions) and prosodically, the “m” in line 13 being short and downward pitch as a mid-turn support, while the “mm” in line 15 is longer and slightly less downward pitch, suggesting acknowledgement as a second action, but still continuer and not co-conclusion as the main action (cf. Sbertoli-Nielsen, 2023). The second premise is that this accounting work is co-achieved as precisely that—as understanding, and not as an excuse^5^ (lines 17–18). The interviewer’s “m” in line 19, is prosodically flat and outdrawn to suggest continuer rather than stance-display as main action. Providing a continuer as opposed to protest or repair work, works to accept this premise (Robinson, 2014) and thus acknowledges Kristoffer as a morally competent member of society. Research from medical settings has shown the importance of gradually establishing a ‘problem context’ before asking problem-oriented questions (Iversen et al., 2017; Pilnick and Coleman, 2003). Similarly, the work Kristoffer and the interviewer do here can be seen as establishing a ‘vulnerability context’ in which it is possible for Kristoffer to start disclosing what he fears others will not understand.

In these last turns of the first phase, Kristoffer concludes his preparation for telling by explaining (lines 20–23) and concluding (lines 25–26): there is a backstory that can make it easier (for “them”) to understand his offenses. To this end, the interviewer employs a range of resources to do *relationally scaffolded storytelling*, which will be addressed in the next section. The interviewer’s subsequent solicitation of disclosure in line 29 (after a silence allowing Kristoffer space to take the floor) maintains this quality of their momentary relationship. While direct, the question respects Kristoffer’s deontic autonomy (Stivers, 2022) by displaying low deontic authority to ask relative to her role as the interviewer, being designed as aligning with his project (connecting it to their previous talk with the inference marker “da”). The subtle use of prosodic resources (soft, low-volume voice) (Couper-Kuhlen, 2012; Kykyri et al., 2017; Weiste and Peräkylä, 2014), works to affiliate without being specific about what she affiliates with (Iversen et al., 2024; Todd-Kvam et al., 2025). This displays an empathic orientation to the emotional weight of Kristoffer’s task of telling, without unduly impinging on his epistemic domain by claiming epistemic access to the experience he is telling her about. This contrasts with the interviewer’s direct question to Hans in Extract 3, which misaligns with his project and thus works to overstep his boundaries.

In sum, the approaching telling phase shows how the participants both orient and actively work to achieve respectful closeness between them while (and even by) addressing threats to it. Kristoffer invites the interviewer to take part in his concern over the potential rejection by “the others,” thereby allowing her response to matter to him and risking rejection in the here-and-now of the interview. This is in stark contrast to Extract 1, where Tord’s turns of talk work to de-weaponize the interviewer, and others in general, by rejecting their opinions and projects as insignificant to him. By being willing to stay with what is difficult for Kristoffer without rejecting him or trying to change it, the interviewer respects Kristoffer’s right to have this experience, co-achieves the nature of their momentary relationship as close (or close enough to talk about these things), and thus communicates acceptance. This co-achieved quality of respectful closeness prepares the ground for commencing disclosure.

#### 4.2.2 Commencing disclosure: relationally scaffolded storytelling

In this phase, the conversation changes from negotiating how to understand Kristoffer’s storytelling (not as excusing, but as explaining to avoid rejection) to actually telling. Storytelling, as a conversational activity, leaves the participants with less maneuvering space to *do* their relationship as close—among other things because the interviewer’s responses are mostly limited to continuers or listening tokens (as opposed to whole turns, cf. Kupetz, 2014; Stivers, 2008), and because the participants’ ability to build a shared understanding is limited by the interviewer’s lack of epistemic access to what the interviewee tells. Moreover, while storytelling in everyday conversations makes relevant recipient responses orienting to (and preferably endorsing) the storyteller’s stance (Stivers, 2008), interviewers might be constrained by ideals of neutrality in how they can answer (Jol and Stommel, 2021; Rapley, 2001). Here, we highlight one phenomenon the interviewer and Kristoffer nonetheless achieve within these constraints: *relationally scaffolded storytelling*. It recurs throughout the entire sequence but is very clearly introduced here by Kristoffer trying out the relationship. We come in immediately after the interviewer’s question in line 29 above. The one-second silence (line 30) indicates that answering is not straightforward for Kristoffer.

**Extract 4.2: Commencing disclosure**30			(1.0)
31	IE:	no well I (.) e:h (.) presented myself as a:
32		girl,
33	IR: 	m:_ (*GAT-2: `m:_)*34	IE: 	e::h (1.5) a::nd (.) thus contacted (1.0)
35		boys of ↓all ↓ages,
36	IR:	°m:_° (*GAT-2: °`m:_°)*37	IE:	and I noticed that (.) if I went out as
38		 (.) myself,
39	IR:	yes_  (*GAT-2:* ˉ *ja_)*40	IE:	then nobody would talk with me.
41	IR: 	°no.° (*GAT-2: °`nei.°*)
42	IE:	because I was a boy.
43	IR: 	°yes° (*GAT-2: °ja°)*44		(.)
45	IE: 	so I went out as a girl_
46	IR: 	°m:_° (*GAT-2: °`m:_°*)
47	IE:	and then I got answers.
48	IR:	°m:_° (*GAT-2: °`m:_°*)
49		(1.0)

Kristoffer’s subsequent turn (lines 34–35) is perturbated and can, like his introduction of the disclosure above (Extract 4a, lines 1–5), be understood as treating the subject as requiring careful management (Silverman and Peräkylä, 1990) and displaying emotional discomfort (Retzinger, 1995). Both these interpretations can be understood as a way of relationally managing a potential rejection (Gausel, 2013; Gausel, 2023). Furthermore, Kristoffer produces the last two words (“all ages,” line 35) with a markedly lower pitch. In the context of being offered in an interview about sexual violations, this information projects particularly problematic content to come, but nonetheless leaves it unspecified. By projecting trouble, he can be heard as displaying vulnerability to rejection. As such, we understand this work as trying out the relationship (Behnia, 2008).

The interviewer’s response to Kristoffer’s treatment of the topic as requiring careful management and to his projection of trouble (line 36) keeps doing continuer work (like her preceding response), but changes its prosodic features to also affiliate with Kristoffer’s affective display (i.e., softly spoken, lower volume; Kykyri et al., 2017; Weiste and Peräkylä, 2014). Moreover, by *not* orienting to the topic as requiring careful management, the interviewer misaligns with Kristoffer’s marking of the content of his disclosure as socially dispreferred to address. This combination of validating his emotional experience through affiliation while misaligning to his treatment of the topic as socially dispreferred to address (i.e., by not joining in) can be understood as therapeutic misalignment (Peräkylä, 2011; Scarvaglieri, 2020). It works to create exceptional interactional space for “that sort of thing to be said here,” thus supporting continuation toward Kristoffer’s projected trouble.

The subsequent dialogue (lines 37–46), like the dialogue in Extract 4a, is marked by Kristoffer’s turns of talk frequently making interviewer responses relevant (short turns, turn-final upward intonation), and the interviewer’s minimal responses responsively varying between affiliation and alignment.

Both here and in Extract 4a, Kristoffer’s turns of talk change in terms of what action they are doing. In Extract 4a, they changed from explaining (lines 20–23: “there was a lot that happened”) to concluding (lines 25–26: “easier for them to understand”); here, they change from doing disclosure (up until line 35) to explanation (lines 37–42: “if I went out as myself”) and back to disclosing (line 45: “so I went out as a girl”).

Through variation in choice of words and prosodic design, the interviewer’s listening tokens responsively *align* with Kristoffer’s changing actions. In Extract 4a, they change from a soft-spoken continuer response during his explanation (line 22) to a downward intonation receipt token (line 24), and to an alignment to his action of concluding with a clearer downward intonation, clearly articulated as “eyeah.” (line 27). In Extract 4b they change from lexically appropriate responses to the elements of his explanations (lines 41 and 43) to continuers supporting his disclosure (lines 46 and 48).

At the same time as *aligning* responsively, the interviewer’s responses employ a range of prosodic features to *affiliate* responsively. First, her voice quality softens and its volume lowers as an affiliative response to Kristoffer’s references to his own victimization history (lines 41, 43, 46, 48) and his perturbated turn design as displaying emotional difficulty (Extract 4a, lines 22, 24). Second, by responding more frequently, as subtly made relevant by Kristoffer, the interviewer’s responses communicate a continued presence with him while he addresses difficult topics. Finally, she uses time as a resource to be close (Couper-Kuhlen, 2012): responding with immediacy to Kristoffer’s display of discomfort (Extract 4a, lines 20–24). In response to the constraints that both storytelling as an activity and the institutional setting place on her participation in the conversation, the interviewer “amps up” the use of prosodic resources in her minimal responses to both align and affiliate responsively. In addition to amplifying the message “I am with you” (Lindström and Sorjonen, 2013, p. 355), the non-specific nature of prosodic resources enables the interviewer to display affiliation without risking being held accountable for affiliating with an inapposite stance (such as excuse-making) or with an experience they cannot claim to know (Couper-Kuhlen, 2012; Kupetz, 2014; Stivers et al., 2023).

We understand both parties’ interactional work as co-achieving *relationally scaffolded storytelling*. Kristoffer solicits extra support while he tells the story about what he fears “others” will not understand (or accept), and the interviewer’s skillful and responsive use of prosodically and lexically varied listening responses actively communicates her continued presence with him, on his terms, throughout the telling. As such, relationally scaffolded storytelling is a way of achieving a respectfully close relationship. Relationally scaffolded storytelling is absent in all three cases of disruption, be it due to the interviewee’s lack of invitation (Tord), the interviewer’s lack of responsive listening (Adam), or the interviewer and interviewee’s project misalignment (Hans).

#### 4.2.3 Returning to understandability: managing absence with presence

In this subsequent phase, Kristoffer leaves his commenced disclosure and returns to addressing the understandability of his offenses. We foreground one phenomenon: how the interviewer *manages absence with presence*. In the first lines (50–63), Kristoffer makes relevant the interviewer’s collaborative storytelling, among other things by treating it as an unfinished project (line 59: “started wondering”), phrasing it as questions (lines 60–68), and inviting feedback using “or?” (Lindström, 1999). Like in the contrasting example above (Adam, Extract 2), the interviewer is put in a position where aligning with Kristoffer’s collaborative telling would disrespect Kristoffer’s right to know and define his story and would challenge the interviewer’s neutralistic stance. Not aligning, on the other hand, involves relationally managing the absence of alignment:

**Extract 4.3a: Returning to understandability**50	IE:	a::nd (.) ↑really quite a lo- I have been thinking
51		(.) a fair share that part of what happened to
52		me, (.) I have done with others.
53		(.)
54	IR:	°eyeah.° (*GAT-2: `°eja.°)*55	IE:	so: (.)i-it is really quite a mirror image_
56	IR: 	yes. (*GAT-2: `ja*.)
57		(.)
58	IR: 	.hyeah_ (*GAT-2: ´.hja_)*59	IE:	so I have you know kind of started wondering
60		a bit about that (.) e:h m e:h (.) Am I trying
61		to recreate something,=have I(.) e:h to- to
62		get rid of my own emotions do I want to transfer
63		them onto others [or?]
64	IR: 		              [eyeah.]	(*GAT-2: ` eja*.)
65			(.)
66	IR:	yes. (*GAT-2: `ja.)*67	IE: 	e::hm (.) is it some kind of revenge because
68		I was subjected to something?=
69	IR: 	°=mm_° (*GAT-2: °=`mm_°*)
70			(.)
71	IE: 	“why does he get to be this ok?” (.) I
72		might as well subject him to it
73	IR: 	.hyeah (*GAT-2: ˉ.hja_)*74	IE:	so that he can take a bit of my (.) burden
75		*da*,^a^76	IR:	yes. right. (*GAT-2: `ja. `Nettopp.*)^b^^a^Norwegian particle here used as inference marker. Original language: «så at han kan ta en del a min (.) belastning da,»^b^Original language: “Ja.Nettopp.” Nettopp can also be translated as “precisely” or “that’s it” depending on context, but “right” best mirrors the meaning here.

The interviewer’s responses (lines 64–66) indeed do not orient to the relevance Kristoffer’s turn sets up, but treat it as an “interview answer” she can acknowledge and receive (Antaki et al., 2000; Houtkoop-Steenstra, 2000). In everyday conversations, the preferred response to a question is to answer (Pomerantz and Heritage, 2013), and at the very least to “do dispreference”: to orient to one’s lack of answer in a way that works to nonetheless preserve the relationship. Accordingly treating her response as insufficient, Kristoffer continues to pursue collaborative telling with another question (lines 67–68). The interviewer keeps refraining from aligning, but in contrast to the extract with Adam above (Extract 2), she here designs her minimal responses using non-verbal resources to *simultaneously* do their momentary relationship as emotionally close. Her frequent listening responses throughout, and use of multiple prosodic resources (soft voice and immediacy; Couper-Kuhlen, 2012) in lines 54 and 69 intensifies her non-verbal affiliation (cf. Ogden, 2012). Upon Kristoffer’s continued pursuit (lines 71–72), she uses the ambiguous nature of “yes” (73: .hja)—which can be used both to agree and to acknowledge (Gardner, 1997)—as a resource to not explicitly misalign.

Thus far, we have seen how the interviewer and Kristoffer face potential relational trouble when he pursues a conversational activity she cannot align with, threatening to disrupt their “doing together” and by implication even rejecting Kristoffer’s treatment of his experience as possible to understand (Heritage, 2011). In response, the interviewer uses a range of prosodic resources to convey their momentary relationship as emotionally close: she manages absence with presence. In the remainder of this phase, she upgrades this presence, even after they realign on Kristoffer’s action of concluding (lines 77–78):

**Extract 4.3b: Returning to understandability, continued**77	IE:	So I see that many of the crimes that c-
78		committed (.) by me,=
79	IR:	=.hyeah (*GAT-2: ´.hja_*)
80	IE:	(.) I have also experienced myself.
81	IR:	°↓yes.° (GAT-2:  	°↓`ja.°)
82			(.)
83	IR:	°there is a connection there kind of?°
84	IE: 	mm, *(GAT-2: ^ˇ^mm,)*85	IR:	°↓mm,° *(GAT-2: < <p>>↓^ˇ^mm,>)*

The interviewer’s response (lines 79, 81) not only aligns but also affiliates with the emotional content of Kristoffer’s conclusion using immediacy and soft voice (Couper-Kuhlen, 2012; Kykyri et al., 2017; Weiste and Peräkylä, 2014). She then upgrades this doing of their momentary relationship as close by providing non-evaluative project recognition (line 83): a mirroring formulation that works to display her recognition of Kristoffer’s previous turns as part of his larger project of making his offenses understandable (for similar examples, see Todd-Kvam et al., 2025). By aligning while withholding evaluation, she treats this sensemaking as positioned within his epistemic and deontic domain (not for her to assess, nor to permit or steer), respecting his autonomy (Stivers, 2022). At the same time, she produces the turn with a soft voice, affiliating with the emotional weight of Kristoffer’s conclusion, thus doing their momentary relationship as close.

This is in contrast to the examples of both Adam and Hans above (Extracts 2 and 3), where the interviewer’s responses refrain from recognizing Adam’s candidate answers as part of an overarching sensemaking project and fail to respect Hans’ epistemic and deontic autonomy. The immediate consequence in both these extracts is disrupted disclosure.

In the present extract, the immediate conversational outcome is an empathic moment (lines 84–85; see Heritage, 2011). Here, Kristoffer’s response to the interviewer’s non-evaluative project recognition (line 84) has an intonation curve signaling agreement (Sbertoli-Nielsen, 2023), thus marking the end of his pursuit. The interviewer’s subsequent response (85) matches this intonation curve, using a softer, lower pitch voice. As such, it aligns with Kristoffer’s action of concluding, affiliates with the emotional weight of his project, and, by doing nothing else, stays with him in it. In line with the institutional setting both participants are oriented to Kristoffer’s emotional experience *as his* (Heritage, 2011; Ruusuvuori, 2005). As we shall see in the next section, this enables Kristoffer to return to disclosure and to eventually answer the interviewer’s question from back in line 29.

#### 4.2.4 Arriving at the answer: more relationally scaffolded storytelling and co-conclusion

Kristoffer marks the return to disclosure by picking up where he started last time—his pretending to be a girl online:

**Extract 4.4: Arriving at the answer**86	IE:	so mainly (.) e:h I presented myself
87		as a girl
88	IR:	yes (*GAT-2: `ja;*)
89	IE:	a:nd (2.0) -i-i-it resulted in (.) a certain
90		period where: (.) after I had been subjected
91		to this (1.0) to continue this then figuring
92		out who I am,
93	IR:	°m:_° (*GAT-2: °`m:_°*)
94	IE: 	 then I slept with extremely many,
95	IR:	yes. (*GAT-2: `ja.*)
96	IE:	so I became:  (.) quite numb kind of
97		(.) sex and sexuality and who I slept with a::nd,
98	IR: 	eyeah_ (*GAT-2: `eja_*)
99	IE:	so within there then I slept with (.) ↑((anonymized))^a^ who were
100		under ↓sixteen.
101	IR:	eyeah. (*GAT-2: `eja.*)
102		(.)
103	IR: 	°.hyeah.°  (*GAT-2: °`.hja.°*)^a^A general noun indicating number of victim(s).

In the subsequent lines (88–97), Kristoffer and the interviewer co-achieve relationally scaffolded storytelling: he invites frequent responses, and her listening tokens both responsively align with his actions and draw on prosodic resources such as frequency, timing and soft voice to achieve affiliation. They are designed as continuers in response to his telling (lines 89–93) and to acknowledge his conclusions (lines 96–100: both his turns start with the inference marker “so,” and her responses use the ambiguity of “yeah” to acknowledge without making a second assessment). Rather than co-concluding with his turns, particularly in response to his projected continuation (“a.nd”) in line 97, her acknowledgement works to treat his turn as unfinished, leaving space for his eventual answer to the question in line 29 (“what was it that you did”). In lines 99–100, he eventually answers: he slept with “((anonymized)) under 16.” While this answer is both vague and mitigated (his crimes are placed within a period of numbness), it is still an act of telling another person something for which you fear they will reject you (cf. Extract 4.2.1). The interviewer’s response aligns with it as an action of concluding (line 101) and, after a short silence, affiliates with the emotional weight of telling her this, right here and right now (line 103: soft-spoken, low volume). As such, they both work to mark it as an answer to her question in line 29, concluding this longer sequence.

Finally, throughout Extracts 4.2.1–4.2.4, the participants co-achieve an overarching dynamic we describe as *mutual trust*. Kristoffer continues to display trust by displaying vulnerability (Behnia, 2008): he orients to the interviewer’s opinion as important and solicits her support during disclosure. The interviewer shows trust in Kristoffer by following his conversational lead through his approaching telling, commencing disclosure, a return to understandability, and the eventual arrival at the answer, even though his road to the answer is both long and takes some turns that could be understood as excusing or digressing rather than sensemaking (Todd-Kvam et al., 2025). Together, these practices and dynamics build a relational robustness enabling Kristoffer to disclose.

## 5 Discussion

With this article, we have demonstrated how relationship quality, while an evasive object of analysis, can be unpacked as an interactional achievement. We have shown how two people build and maintain a relational quality of respectful closeness within the constraints of the interview setting. This respectful closeness is analytically meaningful because it enables them to address a topic both morally sensitive in general and observably treated as threatening the relationship (Extract 4.1).

We identify one overarching dynamic and three practices. Kristoffer’s willingness to display vulnerability and the interviewer’s willingness to follow Kristoffer’s interactional lead characterize an overarching dynamic of *mutual trust*. Kristoffer’s testing of the relationship and the interviewer’s therapeutically misaligning response (Extract 4.2), how Kristoffer frequently makes relevant relational support and the interviewer responsively provides this relational support by displaying continued presence though alignment and affiliation (throughout extract) characterize the practice of *relationally scaffolded storytelling*. The interviewer’s conveyed willingness to contain and stay with Kristoffer’s display of vulnerability (Extract 4.1) and difficulty characterize the practice of *staying with difficulty*. Finally, the interviewer’s increased display of affiliation in sequential positions where she was institutionally constrained from aligning (Extract 4.3) characterize the practice of managing *absence with presence*. Notably, throughout these practices and dynamic, the interviewer relies almost exclusively on minimal listening responses. While possibly resulting from the constraints of the interviewer role and the interview’s agenda, our analysis unpacks the interactional significance of minimal listening responses. Through skillful use of fine-grained prosodic and lexical variation of her listening tokens’ design, the interviewer aligns and affiliates when relevant and possible, in a way that conveys respect for Kristoffer’s epistemic boundaries (not claiming to know his experience) and emotional boundaries (right to display his emotions). This responsive orientation to the nuances of Kristoffer’s ongoing talk achieves respectful closeness: staying immediately close while respecting his autonomy. The contrast examples demonstrate why these nuanced changes in listening responses are analytically meaningful. Moreover, they demonstrate how relationship quality, although built over the course of multiple turns, also is uniquely done—and potentially undone—anew in every turn.

One overarching observation in our analysis is *continuity across variation*: how participants use a range of verbal and vocal resources within different domains of interaction (cf. Stevanovic and Peräkylä, 2014), which all work to do respectful closeness in concert. Within one turn, actions from different domains interact: in Extract 4.3, for instance, the non-specific nature of prosodic resources allowed the interviewer to affiliate with Kristoffer’s emotional stance without claiming to know his experience (thus unduly impinging on his epistemic autonomy). In Extract 4.2, the interviewer intensified affiliation prosodically when alignment was institutionally out of bounds, thus using one dimension to balance out another.

Another key observation concerned continuity across variation *over time*. The participants’ continued achievement of respectful closeness, turn-by-turn and without the disruptions found in our contrastive examples, enabled them to develop “reciprocal expectations of action and understanding” (Deppermann and Pekarek Doehler, 2021, p. 131), of respect and acceptance. Over time and across resources and practices, participants thus built and maintained something contingent on, constituted by, and constitutive of their turn-by-turn interaction: *a relational robustness*, a mutual expectation that it is *possible* both to understand and to show understanding, when facing a topic risking the opposite.

### 5.1 Contributions to theory and research on the relationship as interactionally achieved

Speaking to interactional and clinical literature, our findings may most aptly be captured in the term solidarity (Clayman, 2002; Heritage, 1984; Lindström and Sorjonen, 2013; Pino, 2022). *Respectful closeness* resonates with theoretical conceptualizations of solidarity as a social bond characterized by neither too much distance nor breaching the other’s boundaries (Scheff, 1986). The clinical ideal of unconditional acceptance (Rogers, 1961) connects to this solidary quality: accept is truly unconditional when combined with respect for the other’s autonomy. Our analysis specifies and expands these clinical-theoretical conceptualizations, by demonstrating how they are interactionally achieved. This interconnection between autonomy and closeness is further illustrated by the contrastive examples. The interviewer’s respect for Kristoffer’s autonomy enables their closeness—without it the closeness becomes overstepped boundaries (see Extract 3). Similarly, closeness makes autonomy work. The interviewer’s continued affiliative presence as Kristoffer addressed the emotional and interpersonal difficulties of approaching disclosure enabled her to respect his autonomy in setting the parameters of their interactional space to do so. Extracts 1 and 2 demonstrate how autonomy without this kind of closeness becomes distance. Our analysis thus shows how connection and respect for boundaries are not endpoints on *one* continuum, but *done simultaneously, enabling each other*.

Speaking to interview research specifically, our analysis expands current focus on listening responses’ role in managing neutrality and non-confrontation (cf. Todd-Kvam et al., 2025; Zhang and Okazawa, 2023), to demonstrate the active *supportive* potential of listening responses even within the constraints of the interviewer role. In our analysis, the interviewer was able to *build* relationship (and not just avoid rupture) by responsively adapting her minimal listening tokens to Kristoffer’s various interactional projects.

In analyzing how the participants interactionally achieve respectful closeness, we therefore unpack the interviewer’s *expertise* as a practical skill (cf. Arminen and Simonen, 2021): how to do interviews addressing peoples’ moral transgressions. In this data, expertise is observable as fine-grained *responsivity* (Norcross and Wampold, 2018) to Kristoffer’s ongoing talk, and in the tacit way she accomplishes this responsivity with appropriate minimal listening tokens *at* sequential positions and *in* ways that were possible within institutional and interactional constraints. It demonstrates how she both understands the implications of Kristoffer’s actions (cf. Arminen and Simonen, 2021), and *manages* them: through her responses, the interviewer demonstrates how relationship building has a tacit dimension that is not, and cannot be the same as, the explicit dimension—and must be managed with appropriate, practical (as opposed to propositional) knowledge (Polanyi, 2009). What this tacit responsivity does is show, not tell: she does not *claim* accept in response to Kristoffer’s displayed fear of rejection, by saying something like “I am not here to judge you”. Rather, she *demonstrates* (Enfield, 2011) it continuously, throughout the extract, as secondary actions while doing “listening” as her main action. Accept—trust that one will not be rejected—is built over time, through actions, not just words. The interviewer’s skillful use of minimal responses does not just demonstrate knowing *that* it is so, but *how* to build this relational robustness.

Finally, our analysis goes beyond shared perception or “common ground”- conceptualization of intersubjectivity (cf. Robinson, 2014; Stevanovic and Peräkylä, 2014), to also unpack how the participants interact as two socially situated *subjects*—each with their own autonomy and agency in the relationship, interacting with one another as well as with generalized ideas of the Other (i.e., intersubjectivity as theorized by Benjamin, 2004; Marková, 2003).

### 5.2 Clinical practice implications

Our analysis predominantly examines one single sequence of talk, from a research interview setting, limiting how we may discuss clinical practice implications. Below, we nonetheless argue for our findings’ relevance to clinical practice based on two assertions: first, while taken from a research setting, the analyzed process is profoundly clinical; second, while not directly transferrable, the detailed level of analysis unpacks practices that may gain clinical relevance by spurring reflection and “sensitizing the therapist to their use and role in therapy” (Madill et al., 2001, p. 431).

Our analysis explicates the fundamental relational work in achieving a task that is “simple, but not easy” (Brewer and Giommi, 2025, p. 8). Even in an interactional setting explicitly devoted to interviewees’ disclosure of sexual violations, the participants made a considerable interactional effort to build a relationship in which that disclosure could happen. We discuss three implications.

First, the interviewer’s *doing* of unconditional acceptance emphasizes the central and in itself effective nature of an open, curious, non-judgmental attitude (Brewer and Giommi, 2025; Rogers, 1961) and respectful, attuned responsivity to the client’s expressions of emotion and agency (Norcross and Wampold, 2018). This non-evaluative, interviewee-oriented stance profoundly changes the interactional project. While everyday conversation often orients toward a social project of *agreeing* (Pomerantz and Heritage, 2013)—an interactional project where the participants adjust to each other to find common ground—the interviewer’s stance here builds an interactional project of *allowing and enabling* Kristoffer to simply say and feel what he needs, regardless of the interviewer’s (dis)agreement or evaluation. She treats Kristoffer’s experience as his to have, his to evoke, and his to understand, and not for her to evaluate or change. This is relevant for and beyond the task of facilitating disclosure in a research interview: by successfully demonstrating this attitude, therapists can free their *own* attention from making judgments to allow curiosity and free *the client’s* attention from threat of judgment to exploring causes of their distress.

Here, the idea of “simple, but not easy” gains momentum: achieving a non-judgmental attitude, especially in emotionally potent topics such as sexual violence perpetration, requires continuous self-awareness and self-regulatory skills. This pinpoints the importance of therapists engaging in self-reflective processes to manage potential own issues, such as rigidity and attentional bias (Brewer and Giommi, 2025) or insecure attachment style (Giannotti et al., 2025).

Second, our interactional analysis makes evident that relational work cannot be taught as an abstract theoretical concept to be *applied* in a therapeutic process. Even fundamental therapist skills and attitudes such as unconditional acceptance are achieved in interaction with an agentic, autonomous other. For instance, the effect of the interviewer’s non-evaluative project recognition relied on Kristoffer’s recognition and acceptance of it as such (see Extract 4.3). By comparing the interviewer’s interactional choices in a successful case, compared to contrastive interviewer choices in unsuccessful cases, our analysis exemplifies how we can identify expertise as evaluable, situated actions (Arminen and Simonen, 2021). Our analysis therefore showcases conversation analysis’ capacity to identify and unpack real interactional challenges and their management. This offers a potential bridge over the research-practice gap, by using research findings as a tool to develop clinical expertise as reflexive, relational skills (cf. Stokoe, 2014).

Finally, our findings demonstrate the importance of time and therapist responsivity particularly for detecting and preventing sexual violence. In many institutional settings, clients may be motivated to avoid disclosure because of fear of social and legal sanctions. However, many are also motivated to seek help, and do so for other issues (Lindegren, 2025). The relational quality of respectful closeness we have shown here can support the client’s capacity and willingness to let motives to seek help take precedence over motives to avoid.

### 5.3 Methodological strengths and limitations

Despite representing patterns in the material as a whole and being supported by contrastive cases, our presented analysis is a case study, with caveats for the robustness and transferability of our findings. The participants’ co-creation of respectful closeness exists in and through their doing of it (Garfinkel, 1967); the interplay of practices and resources used to achieve it cannot be directly applied to another local, interactional setting. An interesting avenue for further research would be to examine these practices as distinct phenomena—how they vary and are nuanced across a collection of instances. Moreover, because of its focus on talk as action, our analysis does not consider other aspects relevant to the constitution of meaning in interaction, such as participants’ experiences and socio-cultural context. By focusing on what is observable to both participants as the interview unfolds, however, our analysis demonstrates how we may approach dyadic data with an *inter*active and *inter*subjective focus. By examining the quality of participants’ ongoing relationship as a shared perception, co-achieved in interaction, our study showcases the usefulness of conversation analysis in psychology research (cf. Madill et al., 2001; Muntigl and Scarvaglieri, 2023).

Our overarching observations speak to the importance of complementing collection-based conversation analysis with *longitudinal* conversation analysis. While the former more substantially lends itself to transferability across similar contexts, longitudinal conversation analysis has the potential to bring out overarching interactional dynamics and the interplay of different resources and practices in achieving a larger, interactional outcome (also see Kupetz, 2014; Voutilainen et al., 2010a). Our analysis connects multiple resources and practices to one interactional whole: doing respectful closeness. This interactional whole remains an ongoing, local, interactional outcome; we bring overarching relational quality into sight without losing the emic perspective. Within conversation analysis, our study therefore contributes to the ongoing debate on how we may examine the relationship between participants as an interactional outcome (Enfield, 2011, 2013; Stevanovic and Peräkylä, 2014; Stivers, 2022).

## Data Availability

The original contributions presented in the study are included in the article/supplementary material, further inquiries can be directed to the corresponding author. Due to the sensitivity of the data, the authors are not able to shared further data than what is included in the article.

## Footnotes

1
https://www.nkvts.no/english/report/nesten-innafor-seksuell-vold-og​-​krenkelser-i-unges-naere-relasjoner/

2 We do not provide the full extracts in original language as it would jeopardize interviewee anonymity with such a long extract.

3 We do not provide specific background information on the informant as it would jeopardize their anonymity.

5 “uskyldiggjort” is literally translated as “unguiltymade.”

## References

[ref1] Ahmed E. Harris N. Braithwaite J. Braithwaite V. (2001). Shame Management Through Reintegration. Cambridge: Cambridge University Press.

[ref2] Antaki C. Houtkoop-Steenstra H. Rapley M. (2000). Brilliant. Next question: high-grade assessment sequences in the completion of interactional units. Res. Lang. Soc. Interact. 33, 235–262. doi: 10.1207/s15327973rlsi3303_1

[ref3] Arminen I. Simonen M. (2021). Expertise as a domain in interaction. Discourse Stud. 23, 577–596. doi: 10.1177/14614456211016797

[ref4] Auburn T. (2005). Narrative reflexivity as a repair device for discounting 'cognitive distortions' in sex offender treatment. Discourse Soc. 16, 697–718. doi: 10.1177/0957926505054942

[ref5] Behnia B. (2008). Trust development: a discussion of three approaches and a proposed alternative. Br. J. Soc. Work. 38, 1425–1441. doi: 10.1093/bjsw/bcm053

[ref6] Benjamin J. (2004). Beyond doer and done to: an intersubjective view of thirdness. Psychoanal. Q. 73, 5–46. doi: 10.1002/j.2167-4086.2004.tb00151.x, 14750464

[ref7] Blagden N. J. Winder B. Thorne K. Gregson M. (2011). No-one in the world would ever wanna speak to me again’: an interpretative phenomenological analysis into convicted sexual offenders' accounts and experiences of maintaining and leaving denial. Psychol. Crime Law 17, 563–585. doi: 10.1080/10683160903397532

[ref8] Braithwaite J. (1989). Crime, Shame and Reintegration. Cambridge: Cambridge University Press.

[ref9] Brewer J. Giommi F. (2025). Psychotherapy as investigation: cultivating curiosity and insight in the therapeutic process. Front. Psychol. 16:1603719. doi: 10.3389/fpsyg.2025.1603719, 40771331 PMC12325357

[ref10] Clayman S. E. (2002). “Sequence and solidarity,” in Advances in Group Processes, eds. Thye S. R. Lawrer E. J., vol. 19 (Bingley: Emerald Group Publishing Limited), 229–253.

[ref11] Couper-Kuhlen E. (2012). “Exploring affiliation in the reception of conversational complaint stories,” in Emotions in Interaction, eds. Peräkylä A. Sorjonen M.-L. (New York: Oxford University Press), 113–146.

[ref12] Couper-Kuhlen E. Barth-Weingarten D. (2011). A system for transcribing talk-in-interaction: GAT 2. Translated and adapted for English by Elizabeth Couper-Kuhlen and Dagmar Barth-Weingarten. Gesprächsforschung 12, 1–51.

[ref13] Deppermann A. Pekarek Doehler S. (2021). Longitudinal conversation analysis-introduction to the special issue. Res. Lang. Soc. Interact. 54, 127–141. doi: 10.1080/08351813.2021.1899707

[ref14] Ellemers N. Pagliaro S. Barreto M. Leach C. W. (2008). Is it better to be moral than smart? The effects of morality and competence norms on the decision to work at group status improvement. J. Pers. Soc. Psychol. 95, 1397–1410. doi: 10.1037/a0012628, 19025291

[ref15] Enfield N. J. (2011). “Sources of asymmetry in human interaction: enchrony, status, knowledge and agency,” in The Morality of Knowledge in Interaction, eds. Stivers T. Monada L. Steensig J. (Cambridge: Cambridge University Press), 285–312.

[ref16] Enfield N. J. (2013). Relationship Thinking: Agency, Enchrony, and Human Sociality. Oxford: Oxford University Press.

[ref17] Etelämäki M. (2021). “Organizing the ‘we’in interaction,” in Intersubjectivity in Action: Studies in Language and Social Interaction, eds. Lindström J. Laury R. Peräkylä A. Sorjonen M.-L. (Amsterdam, Netherlands: John Benjamins Publishing Company), 25–39.

[ref18] Etelämäki M. Heinemann T. Vatanen A. (2021). On affiliation and alignment: non-cooperative uses of anticipatory completions in the context of tellings. Discourse Stud. 23, 726–758. doi: 10.1177/14614456211017407

[ref19] Gardner R. (1997). The conversation object mm: a weak and variable acknowledging token. Res. Lang. Soc. Interact. 30, 131–156. doi: 10.1207/s15327973rlsi3002_2

[ref20] Garfinkel H. (1967). “What is ethnometodology?” in Studies in Ethnomethodology, ed. Garfinkel H. (New Jersey, USA: Prentice Hall), 1–34.

[ref21] Gausel N. (2013). “Self-reform or self-defense? Understanding how people cope with their moral failures by understanding how they appraise and feel about their moral failures,” in Walk of Shame, eds. Moshe M. Corbu N. (Nova Publishers), 191–208.

[ref22] Gausel N. (2023). What is shame? Shame as a relational network of emotion-experience. In Ellemers N. Pagliaro S. Nunspeet F. van (Eds.), Handbook on the Psychology of Morality. Routledge.

[ref23] Gausel N. Leach C. W. (2011). Concern for self‐image and social image in the management of moral failure: rethinking shame. Eur. J. Soc. Psychol., 41, 468–478. doi: 10.1002/ejsp.803

[ref24] Giannotti M. Crittenden P. Landini A. Lambruschi F. (2025). Psychotherapists' self-protective attachment strategies. Front. Psychol. 16:37760. doi: 10.3389/fpsyg.2025.1637760, 41112565 PMC12531060

[ref25] Grönte M. Mattsson T. Plantin L. (2024). Strategies to increase fathers' engagement in child protection investigations due to domestic partner abuse in Sweden. Child Fam. Soc. Work 29, 669–678. doi: 10.1111/cfs.13126

[ref26] Heritage J. (1984). Garfinkel and Ethnomethodology. Cambridge: Polity press.

[ref27] Heritage J. (2011). “Territories of knowledge, territories of experience: empathic moments in interaction,” in The Morality of Knowledge in Conversation, eds. Stivers T. Monada L. Steensig J. (Cambridge: Cambridge University Press), 159–183.

[ref28] Houtkoop-Steenstra H. (2000). Interaction and the Standardized Survey Interview: The Living Questionnaire. Cambridge: Cambridge University Press.

[ref29] Iversen C. (2018). Beyond accessing information: claiming to understand in child social welfare interviews. Br. J. Soc. Psychol. 58, 550–568. doi: 10.1111/bjso.12289, 30431156

[ref30] Iversen C. Broström A. Ulander M. (2017). Balancing task focus and relationship building: asking sleepy patients about traffic risk in treatment initiation consultations. Scand. J. Caring Sci. 31, 895–903. doi: 10.1111/scs.12411, 28439962

[ref31] Iversen C. Flinkfeldt M. Hamed S. (2024). Dealing with callers’ racialised talk in suicide preventive helplines: accomplishing (anti) racism in the context of unconditional support. Sociol. Health Illn. 46, 1547–1586. doi: 10.1111/1467-9566.13789, 38861477

[ref32] Jefferson G. (2004). “Glossary of transcript symbols with an introduction,” in Conversation Analysis: Studies from the First Generation, ed. Lerner G. H. (Philadelphia: John Benjamins), 13–31.

[ref33] Jol G. Stommel W. (2021). The interactional costs of “neutrality” in police interviews with child witnesses. Res. Lang. Soc. Interact. 54, 299–318. doi: 10.1080/08351813.2021.1939532

[ref34] Kruse A. E. Houge A. B. (2025). “En sånn fyr? Om erkjennelse og rehabilitering etter å ha blitt dømt for voldtekt,” in Et voldsfelt i bevegelse: Rettslige virkemidler og institusjonelle ordninger, eds. Skilbrei M.-L. Stefansen K. Dullum J. (Cappelen Damm Forskning), 123–144.

[ref35] Kruse A. E. Skilbrei M.-L. (2024). The monster and the self: Taking on the monstrosity of sexual violations. Punishment & Society 26, 840–859. doi: 10.1177/14624745231221933

[ref36] Kupetz M. (2014). Empathy displays as interactional achievements—multimodal and sequential aspects. J. Pragmat. 61, 4–34. doi: 10.1016/j.pragma.2013.11.006

[ref37] Kykyri V.-L. Karvonen A. Wahlström J. Kaartinen J. Penttonen M. Seikkula J. (2017). Soft prosody and embodied attunement in therapeutic interaction: a multimethod case study of a moment of change. J. Constr. Psychol. 30, 211–234. doi: 10.1080/10720537.2016.1183538

[ref38] Lee E. Bhuyan R. (2013). Negotiating within whiteness in cross-cultural clinical encounters. Soc. Serv. Rev. 87, 98–130. doi: 10.1086/669919

[ref39] Levinson S. C. (2013). “Action formation and ascription,” in The Handbook of Conversation Analysis, eds. Sidnell J. Stivers T. (West Sussex: Wiley-Blackwell), 103–130.

[ref40] Lindegren S. (2025). Help-seeking prior to offence and conviction: experiences of individuals convicted of sexual offences. Nord. J. Criminol. 26, 1–18. doi: 10.18261/njc.26.2.8

[ref41] Lindström A. (1999) Language as Social Action: Grammar, Prosody, and Interaction in Swedish Conversation: Grammatik, Prosodi och Interaktion i Svenska Samtal. Uppsala, phd thesis, Acta Universitatis Upsaliensis.

[ref42] Lindström A. Sorjonen M.-L. (2013). “Affiliation in conversation,” in Handbook of Conversation Analysis, eds. Sidnell J. Stivers T. (John Wiley & Sons), 350–369.

[ref43] Madill A. Widdicombe S. Barkham M. (2001). The potential of conversation analysis for psychotherapy research. Counsel. Psychol. 29, 413–434. doi: 10.1177/0011000001293006

[ref44] Marková I. (2003). Constitution of the self: intersubjectivity and dialogicality. Cult. Psychol. 9, 249–259. doi: 10.1177/1354067x030093006

[ref45] Mullins E. Kirkwood S. (2019). Dams, barriers and beating yourself up: shame in groupwork for addressing sexual offending. J. Soc. Work. Pract. 33, 369–384. doi: 10.1080/02650533.2019.1594735

[ref46] Mullins E. Kirkwood S. (2022). Unpicking social work practice skills: warmth and respect in practice. Qual. Soc. Work. 21, 1063–1083. doi: 10.1177/14733250221124214

[ref47] Muntigl P. (2020). Managing distress over time in psychotherapy: guiding the client in and through intense emotional work. Front. Psychol. 10:3052. doi: 10.3389/fpsyg.2019.03052, 32140117 PMC7042173

[ref48] Muntigl P. Horvath A. O. (2014). The therapeutic relationship in action: how therapists and clients co-manage relational disaffiliation. Psychother. Res. 24, 327–345. doi: 10.1080/10503307.2013.807525, 24716569

[ref49] Muntigl P. Horvath A. O. Bänninger-Huber E. Angus L. (2020). Responding to self-criticism in psychotherapy. Psychother. Res. 30, 800–814. doi: 10.1080/10503307.2019.1686191, 31696779

[ref50] Muntigl P. Knight N. Watkins A. Horvath A. O. Angus L. (2013). Active retreating: person-centered practices to repair disaffiliation in therapy. J. Pragmat. 53, 1–20. doi: 10.1016/j.pragma.2013.03.019

[ref51] Muntigl P. Scarvaglieri C. (2023). Discursive angles on the relationship in psychotherapy. Front. Psychol. 14:1198039. doi: 10.3389/fpsyg.2023.1198039, 37404592 PMC10315671

[ref52] Norcross J. C. Wampold B. E. (2018). A new therapy for each patient: evidence-based relationships and responsiveness. J. Clin. Psychol. 74, 1889–1906. doi: 10.1002/jclp.22678, 30334258

[ref53] Ogden R. (2006). Phonetics and social action in agreements and disagreements. J. Pragmat. 38, 1752–1775. doi: 10.1016/j.pragma.2005.04.011

[ref54] Ogden R. (2012). Making sense of outliers. Phonetica 69, 48–67. doi: 10.1159/000343197, 23172239

[ref55] Pascoe Leahy C. (2022). The afterlife of interviews: explicit ethics and subtle ethics in sensitive or distressing qualitative research. Qual. Res. 22, 777–794. doi: 10.1177/14687941211012924

[ref56] Peräkylä A. (2011). “A psychoanalyst’s reflection on conversation analysis’s contribution to his own therapeutic talk,” in Applied Conversation Analysis: Intervention and Change in Institutional Talk, ed. Antaki C. (Basingstoke: Palgrave Macmillan), 222–242.

[ref57] Pillet-Shore D. (2012). Greeting: displaying stance through prosodic recipient design. Res. Lang. Soc. Interact. 45, 375–398. doi: 10.1080/08351813.2012.724994

[ref58] Pilnick A. Coleman T. (2003). “I’ll give up smoking when you get me better”: patients’ resistance to attempts to problematise smoking in general practice (GP) consultations. Soc. Sci. Med. 57, 135–145. doi: 10.1016/S0277-9536(02)00336-2, 12753822

[ref59] Pino M. (2022). Hurting and blaming: two components in the action formation of complaints about absent parties. Res. Lang. Soc. Interact. 55, 260–278. doi: 10.1080/08351813.2022.2101298

[ref60] Polanyi M. (2009). The Tacit Dimension. Chicago: The University of Chicago Press.

[ref61] Pomerantz A. Heritage J. (2013). “Preference,” in The Handbook in Conversation Analysis, eds. Sidnell J. Stivers T. (West Sussex: Blackwell-Wiley), 210–228.

[ref62] Potter J. Hepburn A. (2005). Qualitative interviews in psychology: problems and possibilities. Qual. Res. Psychol. 2, 281–307. doi: 10.1191/1478088705qp045oa, 19957548

[ref63] Prior M. T. (2018). Accomplishing “rapport” in qualitative research interviews: empathic moments in interaction. Appl. Linguist. Rev. 9, 487–511. doi: 10.1515/applirev-2017-0029

[ref64] Rapley T. J. (2001). The art (fulness) of open-ended interviewing: some considerations on analysing interviews. Qual. Res. 1, 303–323. doi: 10.1177/146879410100100303

[ref65] Retzinger S. M. (1995). Identifying shame and anger in discourse. Am. Behav. Sci. 38, 1104–1113. doi: 10.1177/0002764295038008006

[ref66] Richards K. (2011). Using micro-analysis in interviewer training: ‘continuers’ and interviewer positioning. Appl. Linguist. 32, 95–112. doi: 10.1093/applin/amq040

[ref67] Risjord M. (2015). “Evidence and practical knowledge,” in Exploring Evidence-Based Practice: Debates and Challenges in Nursing, ed. Lipscomb M. (London/New York: Routledge), 76–87.

[ref68] Robinson J. D. (2014). What “what?” tells us about how conversationalists manage intersubjectivity. Res. Lang. Soc. Interact. 47, 109–129. doi: 10.1080/08351813.2014.900214

[ref69] Rogers C. R. (1961). On Becoming a Person: A Therapist's View of Psychotherapy. Boston/New York: Houghton Mifflin Harcourt.

[ref70] Roulston K. (2006). Close encounters of the ‘CA’KIND: a review of literature analysing talk in research interviews. Qual. Res. 6, 515–534. doi: 10.1177/1468794106068021

[ref71] Ruusuvuori J. (2005). “Empathy” and “sympathy” in action: attending to patients' troubles in Finnish homeopathic and general practice consultations. Soc. Psychol. Q. 68, 204–222. doi: 10.1177/019027250506800302

[ref72] Sbertoli-Nielsen P. (2023). “Teasing out 2mm”: bilabial-nasal response particles in Norwegian. Norsk Lingvist. Tidsskr. 41, 207–232.

[ref73] Scarvaglieri C. (2020). First encounters in psychotherapy: relationship-building and the pursuit of institutional goals. Front. Psychol. 11:585038. doi: 10.3389/fpsyg.2020.585038, 33384639 PMC7769839

[ref74] Scheff T. J. (1986). Micro-linguistics and social structure: a theory of social action. Sociol Theory 4, 71–83. doi: 10.2307/202106

[ref75] Schegloff E. A. (2007). Sequence Organization in Interaction: A primer in Conversation Analysis I, vol. 1. Cambridge: Cambridge university press.

[ref76] Sidnell J. Stivers T. (2013). The Handbook of Conversation Analysis. West Sussex: Wiley-Blackwell.

[ref77] Sikveland R. O. (2012). Negotiating towards a next turn: phonetic resources for ‘doing the same’. Lang. Speech 55, 77–98. doi: 10.1177/0023830911428859, 22480027

[ref78] Sikveland R. O. Solem M. S. Skovholt K. (2021). How teachers use prosody to guide students towards an adequate answer. Linguist. Educ. 61:100886. doi: 10.1016/j.linged.2020.100886

[ref79] Silverman D. Peräkylä A. (1990). AIDS counselling: the interactional organisation of talk about ‘delicate’issues. Sociol. Health Illn. 12, 293–318.

[ref80] Steensig J. (2020). “Conversation analysis and affiliation and alignment,” in The Concise Encyclopedia of Applied Linguistics, ed. Chapelle C. A. (West Sussex: Wiley Blackwell), 248–253.

[ref81] Stevanovic M. Peräkylä A. (2014). Three orders in the organization of human action: on the interface between knowledge, power, and emotion in interaction and social relations. Lang. Soc. 43, 185–207. doi: 10.1017/s0047404514000037

[ref82] Stivers T. (2008). Stance, alignment, and affiliation during storytelling: when nodding is a token of affiliation. Res. Lang. Soc. Interact. 41, 31–57. doi: 10.1080/08351810701691123

[ref83] Stivers T. (2022). The Book of Answers: Alignment, Autonomy, and Affiliation in Social Interaction. Oxford: Oxford University Press.

[ref84] Stivers T. Rossi G. Chalfoun A. (2023). Ambiguities in action ascription. Soc. Forces 101, 1552–1579. doi: 10.1093/sf/soac021

[ref85] Stokoe E. (2014). The conversation analytic role-play method (CARM): a method for training communication skills as an alternative to simulated role-play. Res. Lang. Soc. Interact. 47, 255–265. doi: 10.1080/08351813.2014.925663

[ref86] Todd-Kvam M. Gausel N. Överlien C. (2025). Making excuses and making sense: the role and nuances of active listening in eliciting and managing accounts of sexual violations. Qualitative Social Work. doi: 10.1177/14733250251388709

[ref87] Voutilainen L. Peräkylä A. Ruusuvuori J. (2010a). Misalignment as a therapeutic resource. Qual. Res. Psychol. 7, 299–315. doi: 10.1080/14780880902846411

[ref88] Voutilainen L. Peräkylä A. Ruusuvuori J. (2010b). Recognition and interpretation: responding to emotional experience in psychotherapy. Res. Lang. Soc. Interact. 43, 85–107. doi: 10.1080/08351810903474799

[ref7001] Wampold B. E. (2015). How important are the common factors in psychotherapy? An update. World psychiatry, 14, 270–277. doi: 10.1002/wps.20238 PMC4592639 26407772

[ref89] Weiste E. Peräkylä A. (2014). Prosody and empathic communication in psychotherapy interaction. Psychother. Res. 24, 687–701. doi: 10.1080/10503307.2013.879619, 24517281

[ref90] Zhang T. Okazawa R. (2023). Managing neutrality, rapport, and antiracism in qualitative interviews. Qual. Res. 23, 1689–1713. doi: 10.1177/14687941221110183

